# *JACC: Advances*: Committed to Health Equity

**DOI:** 10.1016/j.jacadv.2023.100437

**Published:** 2023-07-28

**Authors:** Paul L. Douglass, Dipti Itchhaporia, Candice K. Silversides

**Figure:**
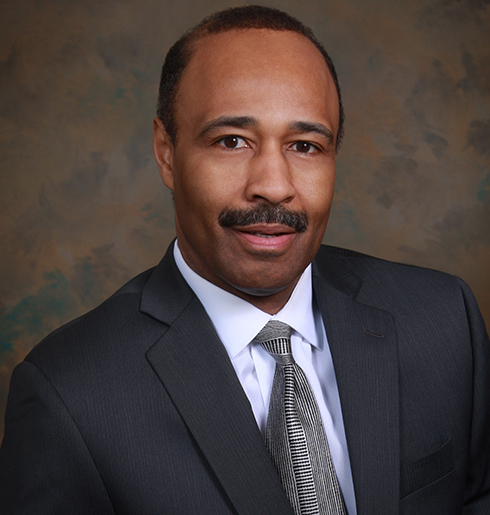

**Figure:**
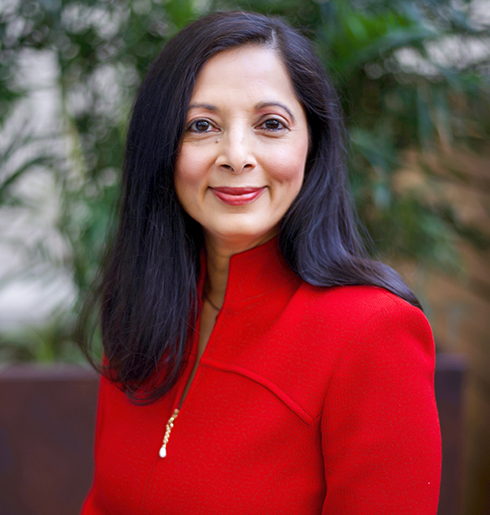

**Figure:**
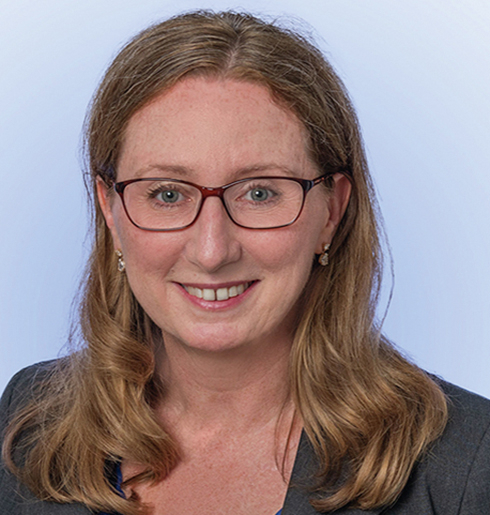

There is growing commitment by many cardiovascular societies to address health care disparities, both locally and globally, and to support ongoing discussions and programs centered on health equity. A large body of evidence shows that the integration of diversity, equity, and inclusion principles can reduce bias, enhance the quality of care, and improve health outcomes. Although factors such as race, ethnicity, and sex are important drivers of cardiovascular disparities, social determinants of health, the conditions in which people are born, work, live, grow, play, and are educated account for 80% of cardiovascular outcomes. Health equity initiatives recognize and account for these individual differences, with the goal of delivering fair and equitable cardiovascular care for all. The American College of Cardiology (ACC) has long recognized the importance of addressing health disparities^1^ and has developed strategic initiatives addressing heath inequities, including the development of a strategic health equity plan, creation of a health equity task force, and supporting the first health equity hub at ACC.23, a health equity roundtable, and a summit in 2023.

The cardiovascular scientific community also has an important role in helping to reduce and eliminate health disparities and inequities. Acknowledgment of the role of the legacy of systemic racism and unjust health policies is key to addressing health inequities. Unfortunately, medical research has contributed to health inequities. Many studies have been too narrowly focused and based on study cohorts that lacked diversity. This has resulted in studies with limited relevance to the population at large, especially in underrepresented groups and minority communities. Furthermore, research specifically addressing health inequities has been underfunded and there has been an underrepresentation of minorities in medical research. Fortunately, there are a number of solutions to help address these issues,^2^ and granting agencies, such as the National Institutes of Health, are also committed to increasing diversity in science.

At *JACC: Advances*, we strive to publish high-quality clinically impactful science and reviews, including those related to health equity. Our editorial board is invested in this topic and includes the current and immediate past chair of the ACC Heath Equity Taskforce, Drs Paul Douglass and Dipti Itchhaporia. We are aware that editorial board composition can play a role in decreasing bias that can contribute to inequities. As such, our editorial board includes many women and members of various ages and racial/ethnic backgrounds and from different geographic locations. This mix helps to ensure that various perspectives are included in the journal and bias is minimized in the review and acceptance process. Health equity is clearly an important topic for our authors. For example, in this issue of *JACC: Advances*, Sevilla-Cazes et al^3^ report on the impact of residential racial segregation on the diagnosis and treatment of aortic stenosis.^3^ Our authors have also published original research papers and viewpoints addressing inequities due to racial and socioeconomic factors,^4^, ^5^, ^6^, ^7^, ^8^, ^9^, ^10^ language barriers^11^ the environment,^12^^,^^13^ and access to cardiovascular surgery.^14^ Furthermore, they have contributed manuscripts addressing inequities in broader topics such as research methodologies,^15^^,^^16^ authorship representation,^17^ and editorial board composition.^18^

While there is still a lot of work to be accomplished, at *JACC: Advances*, we are committed to helping understand and improve health inequities in cardiology, eliminating health disparities, and achieving social justice.
